# Society Meetings

**Published:** 1915-08

**Authors:** 


### SOCIETY MEETINGS

---

Brief reports and announcements of meetings of societies of Western New York, and those of general scope, are requested from Secretaries. Copy should be on hand the fifteenth of the month. Full transactions will be published at cost of composition.

---

**DOCTOR: Is your Society properly represented here? If not, please bring our standing announcement to the attention of the members.**

---

**Lake Keuka Medical and Surgical Association,** embracing the counties of Allegany, Chemung, Erie, Livingston, Monroe, Onondaga, Ontario, Schuyler, Seneca, Steuben, Tioga, Wayne,

Wyoming and Yates, met at Grove Springs Hotel, Keuka Lake, Thursday and Friday, July 22 and 23. Program—President's address, Ross G. Loop, Elmira; "The Old and New in Public Health," B. R. Wakeman, Hornell, discussion opened by William E. Barron, M. D., Addison; "Innocent and Accidental Syphilis," E. Wood Ruggles, Rochester; "Tetanus," John M. Quirk, Montour Falls; "Obstetric Amnesia," William T. Getman, Buffalo; "Prognosis of Hyperthyroidea," Henry L. Elsner, Syracuse, discussion opened by Martin B. Tinker, Ithaca; "Present Status of Physical Therapeutics," John C. Fisher, Elmira, discussion opened by Floyd S. Crego, Buffalo; "The Surgical Treatment of Ulcer and Cancer of the Stomach, Pylorus and Duodenum: How Efficient is it? A. L. Soresi, New York; "Duodenal Ulcer:A Survey of 100 Operative Cases," John F. Erdman, New York; Discussion of papers of Drs. Soresi and Erdmann opened by A. L. Beahan, Canandaigua; "The Function of the Gall-Bladder," Arthur W. Booth, Elmira, discussion opened by Homer J. Knickerbocker, Geneva; "Backache, its Etiology, Diagnosis and Treatment, Roland O. Meisenbach, Buffalo; "The Diagnosis of Intusseption," Irving M. Snow, Buffalo; "The Treatment of Intusseption," Frederick J. Parmenter, Buffalo; Discussion of papers of Drs. Snow and Parmenter opened by Herbert A. Smith, Buffalo; "Diabetes Melitus: Its Etiology and Treatment, John R. Williams, Rochester; "Glycosuria: Its Relation to Surgery," William I. Dean, Rochester, discussion opened by W. W. Skinner, Geneva; "Some Problems of Heredity," Frank L. Christian, Elmira; "Recent Activities of the State Institute for the Study of Malignant Disease," Harvey R. Gaylord, Buffalo; "Exhibit of Bone and Joint Conditions commonly seen by the General Practitioner," Roland O. Meisenbach, Buffalo.

---

**The American Society for Physicians' Study Travels** Contemplates a tour about Sept. 11 from Philadelphia to Baltimore, Old Point Comfort, Richmond, White Sulphur Springs, and Virginia Hot Springs, returning to Philadelphia for the meeting of the Medical Society of the State of Pennsylvania. The total per capita expense will not exceed $40, and the decision rests with the number of favorable responses. Physicians interested are asked to communicate promptly with Dr. Albert Bernheim, Secretary General, 1225 Spruce Street, Philadelphia.

